# Human CD4^+^CD25^+^CD226^-^ Tregs Demonstrate Increased Purity, Lineage Stability, and Suppressive Capacity Versus CD4^+^CD25^+^CD127^lo/-^ Tregs for Adoptive Cell Therapy

**DOI:** 10.3389/fimmu.2022.873560

**Published:** 2022-05-26

**Authors:** Matthew E. Brown, Leeana D. Peters, Seif R. Hanbali, Juan M. Arnoletti, Lindsey K. Sachs, Kayla Q. Nguyen, Emma B. Carpenter, Howard R. Seay, Christopher A. Fuhrman, Amanda L. Posgai, Melanie R. Shapiro, Todd M. Brusko

**Affiliations:** ^1^ Department of Pathology, Immunology and Laboratory Medicine, College of Medicine, Diabetes Institute, University of Florida, Gainesville, FL, United States; ^2^ ROSALIND, Inc., San Diego, CA, United States; ^3^ NanoString Technologies, Inc., Seattle, WA, United States; ^4^ Department of Pediatrics, College of Medicine, Diabetes Institute, University of Florida, Gainesville, FL, United States

**Keywords:** CD226, Treg, lineage stability, suppressive function, autoimmune disease, adoptive cell therapy

## Abstract

Regulatory T cell (Treg) adoptive cell therapy (ACT) represents an emerging strategy for restoring immune tolerance in autoimmune diseases. Tregs are commonly purified using a CD4^+^CD25^+^CD127^lo/-^ gating strategy, which yields a mixed population: 1) cells expressing the transcription factors, FOXP3 and Helios, that canonically define lineage stable thymic Tregs and 2) unstable FOXP3^+^Helios^-^ Tregs. Our prior work identified the autoimmune disease risk-associated locus and costimulatory molecule, CD226, as being highly expressed not only on effector T cells but also, interferon-γ (IFN-γ) producing peripheral Tregs (pTreg). Thus, we sought to determine whether isolating Tregs with a CD4^+^CD25^+^CD226^-^ strategy yields a population with increased purity and suppressive capacity relative to CD4^+^CD25^+^CD127^lo/-^ cells. After 14d of culture, expanded CD4^+^CD25^+^CD226^-^ cells displayed a decreased proportion of pTregs relative to CD4^+^CD25^+^CD127^lo/-^ cells, as measured by FOXP3^+^Helios^-^ expression and the epigenetic signature at the *FOXP3* Treg-specific demethylated region (TSDR). Furthermore, CD226^-^ Tregs exhibited decreased production of the effector cytokines, IFN-γ, TNF, and IL-17A, along with increased expression of the immunoregulatory cytokine, TGF-β1. Lastly, CD226^-^ Tregs demonstrated increased *in vitro* suppressive capacity as compared to their CD127^lo/-^ counterparts. These data suggest that the exclusion of CD226-expressing cells during Treg sorting yields a population with increased purity, lineage stability, and suppressive capabilities, which may benefit Treg ACT for the treatment of autoimmune diseases.

## Introduction

Human regulatory T cells (Tregs) possess the unique capacity to suppress innate and adaptive immune subsets throughout the body using a variety of mechanisms, including consumption of growth factors, degradation of inflammatory substrates, expression of negative regulators of costimulation, secretion of immunoregulatory cytokines, and trogocytosis (1–3). Impairment of Treg suppression *in vivo* leads to the proliferation of autoreactive T cells, which has been associated with the development of autoimmune diseases, such as type 1 diabetes (T1D) and systemic lupus erythematosus (SLE) (4, 5). Therefore, Tregs represent a critical target or even deliverable component of immunotherapies seeking to inhibit the pathogenesis of autoimmune diseases (6, 7).

Early proof-of-principle studies in the non-obese diabetic (NOD) mouse provide evidence that adoptive transfer of Tregs can reverse autoimmune diabetes (8–10). Translating this concept to patients with or at risk for T1D requires the isolation and subsequent *ex vivo* expansion of Tregs for adoptive cell therapy (ACT), due to the rarity of Tregs in both peripheral and umbilical cord blood (6, 11–14). Polyclonal autologous Treg-ACT was shown to be safe yet ineffective at preserving insulin production in individuals with recent-onset T1D (6), potentially due to limited Treg persistence *in vivo*. In a recent phase I clinical trial, low dose IL-2 bolstered polyclonal Treg engraftment in patients with T1D but also, imparted undesirable off-target expansion of cytotoxic cell subsets, such as activated natural killer (NK), mucosal associated invariant T (MAIT), and CD8^+^ T cells (15). Hence, there is a clear need to optimize Treg ACT, including through isolation of a Treg population that maintains lineage stability and suppressive functionality following *ex vivo* expansion.

Early Treg enrichment strategies relied on the observation that Tregs constitutively express the IL-2 receptor alpha chain (IL-2Rα/CD25), conferring a high affinity for the T cell growth factor, IL-2 (16). However, observations of activation-induced upregulation of CD25 on CD4^+^ conventional T cells (Tconv) (17, 18) supported the need for additional markers for effective Treg isolation (19). Current Treg isolation methods involve Fluorescence-Activated Cell Sorting (FACS) of CD4^+^CD25^hi^ T cells with low to no expression of the IL-7 receptor, CD127 (20). However, CD127 can be downregulated by Tconv in response to signaling by IL-7 and other common γ-chain cytokines (20). Moreover, in instances of lymphopenia, increased serum levels of IL-7 are known to decrease CD127 expression on Tconv, significantly complicating efforts to isolate tolerogenic Tregs for ACT in patients with autoimmune diseases (21–23).

The CD127^lo/-^ Treg isolation strategy yields a heterogeneous population containing both lineage stable FOXP3^+^Helios^+^ Tregs as well as FOXP3^+^Helios^-^ Tregs, which are susceptible to phenotypic instability upon activation (24, 25). While subject to debate (26), the FOXP3^+^Helios^+^ transcription factor combination is generally accepted as identifying the thymically-derived Treg subset (tTregs) while FOXP3^+^Helios^-^ designates the peripherally-induced Treg fraction (pTregs) (25, 27). Compared to tTregs, pTregs exhibit increased production of inflammatory cytokines, including IFNγ, as well as methylation at the conserved non-coding sequence 2 (CNS-2), referred to as the Treg-specific demethylation region (TSDR) (28). As a result, the CD4^+^CD25^hi^CD127^lo/-^ population is predisposed to an outgrowth of less suppressive Tregs and increased expression of inflammatory/effector molecules during expansion, all of which may negatively impact ACT therapeutic efficacy (29). Furthermore, an increased proportion of IFNγ-secreting FOXP3^+^Helios^-^ Tregs in patients with T1D versus healthy controls suggests that detrimental Treg plasticity may be augmented in settings of inflammation or autoimmunity (28).

Previous work in our laboratory characterizing the phenotype of IFNγ-secreting FOXP3^+^Helios^-^ Tregs revealed high expression of the costimulatory molecule CD226 (30). CD226 is an activating costimulatory receptor associated with the initiation of Th1 and Th17 immune responses (31, 32). Following its ligation with CD112 or CD155 on antigen-presenting cells (APCs), CD226 becomes activated *via* phosphorylation of its immunoreceptor tyrosine-based activation motif (ITAM) (33), augmenting downstream Ras/MAPK signaling, which is known to result in increased secretion of the pro-inflammatory cytokines IFN-γ and IL-17A (31). In our studies, CD226 expression correlated positively with CD127 and negatively with FOXP3 expression; moreover, freshly isolated CD226^lo^ Tregs exhibited increased demethylation at the TSDR as compared to CD226^+^ Tregs, suggesting high CD226 expression might be associated with an effector phenotype (30).

In addition to contributing to decreased regulatory function, *CD226* has been identified to contain a potential gain-of-function risk variant contributing to a propensity for multiple autoimmune diseases including T1D, SLE, rheumatoid arthritis (RA), and multiple sclerosis (MS) (32, 34–36). We previously reported that knockout (KO) of *Cd226* in NOD mice resulted in reduced severity of insulitis and diabetes incidence (37), and Wang et al. similarly observed that *Cd226* KO reduced disease severity in an experimental autoimmune encephalomyelitis (EAE) mouse model of MS, further highlighting the role of CD226 in autoimmune disease pathogenesis (38).

To identify an improved surrogate surface marker for lineage stable Tregs, we performed extensive *ex vivo* analyses to evaluate the therapeutic potential of CD4^+^CD25^+^CD226^-^ sorted T cells as compared to the conventional CD4^+^CD25^+^CD127^lo/-^ strategy. Specifically, we hypothesized that this marker profile would allow for isolation and expansion of increased proportions of FOXP3^+^Helios^+^ Tregs, minimizing contamination of IFNγ-producing FOXP3^+^Helios^-^ Tregs, to yield a more stable and functionally suppressive population

## Materials and Methods

### Human Subjects

Fresh peripheral blood mononuclear cell (PBMC) samples were isolated from human leukapheresis-enriched blood of healthy donors (median age: 22 years, range 18-39 years, *N*=20, 45% female) purchased from LifeSouth Community Blood Centers (Gainesville, FL).

### CD4^+^ T Cell Enrichment From Human PBMC Samples

Before Treg isolation, CD4^+^ T cells were enriched by negative selection using a CD4^+^ T cell enrichment RosetteSep™ cocktail (StemCell Technologies, Vancouver, BC, Canada) according to the manufacturer’s instructions while autologous PBMCs required for suppression assays were isolated from unenriched peripheral blood. CD4^+^ T cell-enriched and unenriched components were diluted 1:1 with PBS and overlayed onto Ficoll-Paque Plus medium (Thermo Fisher, Waltham, MA, USA) for density gradient centrifugation (1200 x g, 20 min). PBMCs were suspended in Ammonium-Chloride-Potassium (ACK) Lysis Buffer (Gibco, Waltham, MA, USA), washed, and resuspended in PBS, according to the manufacturer’s instructions. Quantification of cell viability was accomplished by staining with Acridine Orange/Propidium Iodide (AO/PI) before reading on an Auto2000 Cellometer (Nexcelom Biosciences, Lawrence, MA, USA).

### FACS Isolation of Treg Subsets

**Figure 1:**
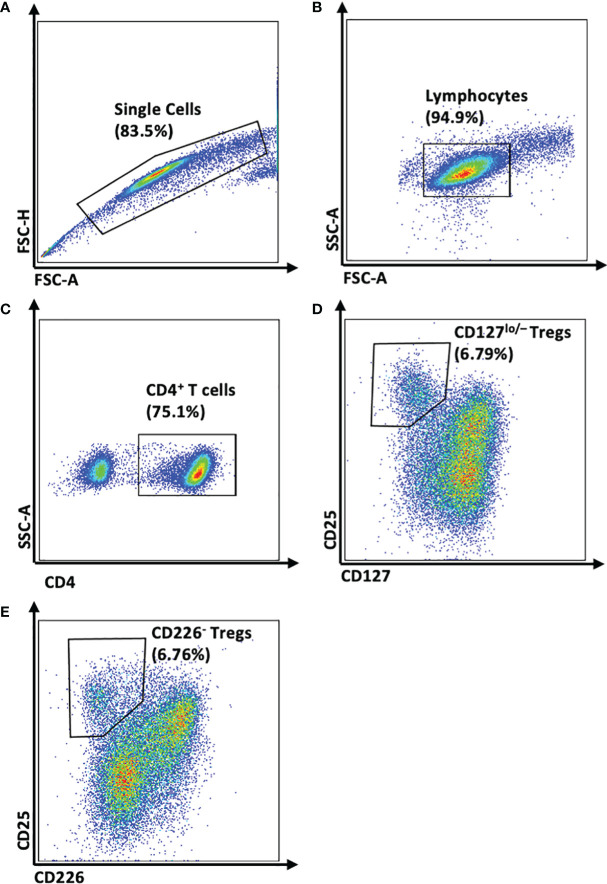
Gating Strategy for FACS Isolation of Paired CD4^+^CD25^+^CD127^lo/-^ and CD4^+^CD25^+^CD226^-^ Tregs. Representative flow plots demonstrate the method by which CD127^lo/-^ or CD226^-^ Tregs were isolated from CD4^+^ T-cell enriched PBMC using a BD FACSAriaIII Cell Sorter. **(A)** Singlet gating was performed using forward scatter area (FSC-A) versus forward scatter height (FSC-H). **(B)** Lymphocytes were gated on FSC-A and side scatter area (SSC-A). **(C)** From the CD4^+^ T cell fraction, **(D)** CD25^+^CD127^lo/-^ Tregs and **(E)** CD25^+^CD226^-^ Tregs were isolated.

CD4^+^ T cell-enriched PBMCs were split and stained with: 1) CD4-BV510, CD25-APC, and CD127-PE or 2) CD4-BV510, CD25-APC, and CD226-PE-Cy7 (clone and manufacturer information provided in **Table 1**). Matched sets of CD4^+^CD25^+^CD127^lo/-^ and CD4^+^CD25^+^CD226^-^ Tregs were isolated (**Figure 1**) using a FACSAria™ III Cell Sorter (Beckton Dickinson, Franklin Lakes, NJ, USA; CD4^+^CD25^+^CD127^lo/-^ median sort purity: 96.1%, range: 85.8-99.9%, *N*=6; CD4^+^CD25^+^CD226^-^ median sort purity: 97.8%, range: 88.7-99.9%, *N*=6).

**Table 1:** Antibodies used for flow cytometry.

Target	Clone	Fluorochrome	Vendor	Concentration	RRID
CD4	SK3	BV510	BD Biosciences	0.05 µg/mL	AB_2744424
CD8	RPA-T8	PE-CF594	BD Biosciences	0.10 µg/mL	AB_11154052
CD25	BC96	APC	BioLegend	0.50 µg/mL	AB_314280
CD25	BC96	BV605	BioLegend	0.50 µg/mL	AB_11218989
CD39	eBioA1	APC	eBioscience	0.50 µg/mL	AB_1963578
CD40L	24-31	APC-Cy7	BioLegend	0.50 µg/mL	AB_2076096
CD45RA	HI100	BV605	BioLegend	0.10 µg/mL	AB_2563814
CD73	AD2	PE	BD Pharmingen	0.50 µg/mL	AB_393561
CD127	A019D5	PE	BioLegend	0.20 µg/mL	AB_1937251
CD197 (CCR7)	2-L1-A	APC-R700	BD Biosciences	0.10 µg/mL	AB_2869856
CD226	11A8	PE-Cy7	BioLegend	0.40 µg/mL	AB_2616645
CLTA-4	L3D10	PE-Cy7	BioLegend	0.50 µg/mL	AB_2563098
FOXP3	206D	Alexa Fluor 488	BioLegend	0.50 µg/mL	AB_430883
FOXP3	259D	Alexa Fluor 488	BioLegend	0.50 µg/mL	AB_430887
GITR	621	PE-Cy5	BioLegend	0.50 µg/mL	AB_2240646
Helios	22F6	Pacific Blue	BioLegend	0.25µg/mL	AB_10690535
IL-10	JES3-9D7	BV421	BioLegend	0.08 µg/mL	AB_2632952
IL-17A	BL168	BV605	BioLegend	0.12 µg/mL	AB_2563887
IFN-γ	4S.B3	BV570	BioLegend	0.10 µg/mL	AB_2563880
PD-1	EH12.2H7	Alexa Fluor 647	BioLegend	0.50 µg/mL	AB_940471
TGF-β1	TW4-2F8	Alexa Fluor 647	BioLegend	0.40 µg/mL	AB_2721298
TGF-β1	FNLAP	PerCP-eFluor 710	eBioscience	0.50 µg/mL	AB_2573900
TNF	Mab11	BV650	BioLegend	0.20 µg/mL	AB_2561355

### Treg Expansion

Following FACS isolation, cells were expanded for 14 days *ex vivo* (13). In brief, Tregs were cultured in complete RPMI media (cRPMI; RPMI 1640 media Phenol Red w/o L-Glutamine (Lonza, Basel, CH-BS, Switzerland), 5mM HEPES (Gibco, Waltham, MA, USA), 5 mM MEM Non-Essential Amino Acids (NEAAs; Gibco), 2mM Glutamax (Gibco), 50 µg/mL penicillin (Gibco), 50 µg/mL streptomycin (Gibco), 20 mM sodium pyruvate (Gibco), 50 mM 2-mercaptoethanol (Sigma-Aldrich, St. Louis, MO, USA), 20 mM sodium hydroxide (Sigma-Aldrich) and 10% FBS (Genesee Scientific, El Cajon, CA, USA)) with Teceleukin recombinant human IL-2 (rhIL-2; Roche, Basel, CH-BS, Switzerland) at 300 IU/mL, with media and rhIL-2 being replaced every 3-4 days. Tregs were stimulated using MACS^®^ GMP ExpAct Treg Beads (Miltenyi Biotec, Bergisch Gladbach, NW, Germany) at a 4:1 bead:cell ratio. Beads were replaced at day seven, and cells were expanded through day 14.

### Analysis of TSDR Epigenetic Signature

Demethylation of the FOXP3-TSDR, or conserved non-coding sequence (CNS2), represents a robust epigenetic indicator of tTreg purity (39). We quantified demethylation within the FOXP3-TSDR by real-time polymerase chain reaction (RT-PCR) as previously described (30), with the following modifications. DNA extraction was conducted using the DNeasy^®^ Blood & Tissue Kit (QIAGEN, Hilden, NW, Germany) as described by the manufacturer’s protocol. Following extraction, DNA was quantified using the Qubit™ Double-Stranded DNA (dsDNA) High Sensitivity (HS) Assay Kit (Invitrogen, Waltham, MA, USA) on the Qubit™ Fluorometer system (Invitrogen). Bisulfite conversion of DNA was conducted using the EZ DNA Methylation™ Kit (Zymo Research, Irvine, CA, USA). RT-PCR was performed using a StepOne™ system (Applied Biosystems, Waltham, MA, USA).

### Flow Cytometric Analysis of Treg Phenotype

To assess the phenotype and purity of Tregs before and following 14 days of expansion, 1 x 10^5^ CD4^+^CD25^+^CD226^-^ and CD4^+^CD25^+^CD127^lo/-^ Tregs were stained with Live/Dead™ Near-IR viability dye (Thermo Fisher) for 10 minutes at 4°C before washing with stain buffer (PBS + 2% FBS + 0.05% NaN_3_ w/v). Cells were then stained with an extracellular antibody cocktail, consisting of CD4-BV510, CD25-APC, CD45RA-BV605, CD127-PE, CD197-APC-R700, and CD226-PE-Cy7 for 30 minutes at 4°C (antibody clone and concentration are provided in **Table 1**). Cells were fixed and permeabilized using the eBioScience™ FOXP3 Transcription Factor Staining Buffer Set (Invitrogen) according to the manufacturer’s instructions, then stained with an intracellular transcription factor antibody cocktail, consisting of FOXP3-Alexa Fluor 488 and Helios-Pacific Blue (**Table 1**). Data were collected on an Aurora 3L (16V-14B-8R) spectral flow cytometer (Cytek, Fremont, CA, USA), and analysis was conducted using FlowJo™ version 10.6.1 Software (BD Life Sciences, Ashland, OR, USA). Tregs were classified as CD4^+^CD25^+^FOXP3^+^, CD4^+^CD25^+^FOXP3^+^Helios^+^, and CD4^+^CD25^+^FOXP3^+^Helios^-^ with phenotype established based on CD45RA and CD197 (CCR7) expression as follows: CD45RA^+^CCR7^+^ naïve, CD45RA^-^CCR7^+^ central memory (T_CM_), CD45RA^-^CCR7^-^ effector memory (T_EM_), and CD45RA^+^CCR7^-^ effector memory re-expressing CD45RA (T_EMRA_) cells. The detailed gating strategy is shown in **Figure S1**. Protein expression levels were reported as stain indices [SI = geometric mean fluorescence intensity (gMFI) of the stained sample/gMFI of the applicable fluorescence minus one (FMO) control].

### Flow Cytometric Analysis of Intracellular Cytokine Production

Following 14 days of *ex vivo* expansion as described above, MACS^®^ GMP ExpAct Treg Beads were removed, then CD4^+^CD25^+^CD127^lo/-^ and CD4^+^CD25^+^CD226^-^ sorted Tregs were immediately assessed for intracellular cytokine expression. Cells were either stimulated with PMA (10 µg/mL; Thermo Fisher) and ionomycin (500 nM; Thermo Fisher) or unstimulated for four hours in the presence of GolgiStop (0.66 µL/mL; BioLegend, San Diego, CA, USA). Stimulated cells underwent staining for viability and extracellular markers, including CD4-BV510, CD25-APC, CD127-PE, CD226-PE-Cy7, and TGF-β1-PerCP-eFluor 710 (**Table 1**), and were subsequently permeabilized as described above. Following permeabilization, cells were stained with the FOXP3-AF488 and Helios-Pacific Blue cocktail, as well as an intracellular cytokine cocktail consisting of IL-10-BV421, IL-17A-BV605, IFN-γ-BV570, TGF-β1-Alexa Fluor 647, and TNF-BV650 (**Table 1**). Fold change of cytokine expression levels were assessed by dividing the gMFI of the stained, stimulated sample by the gMFI of the applicable stained, unstimulated control. Differences between fold change of cytokine expression are reported as Z-scores, [Z = (Mean fold change for Treg subset – Mean fold change for all Tregs assessed)/standard deviation of the sample].

### Flow Cytometric Analysis of Treg Activation Markers

Following 14 days of *ex vivo* expansion, CD4^+^CD25^+^CD127^lo/-^ and CD4^+^CD25^+^CD226^-^ sorted Tregs were labeled with CellTrace™ Violet (CTV; Thermo Fisher) as recommended by the manufacturer, then cultured with no PBMCs or stimulation (0 hour condition) or with autologous PBMCs at a 1:1 ratio in the presence of soluble anti-CD3 (8 µg/mL, Clone OKT3, BioLegend, RRID: AB_11150592) and soluble anti-CD28 (4 µg/mL, Clone CD28.2, Thermo Fisher, RRID: AB_468926) for 24 or 48 hours. Cells were stained for viability with Live/Dead™ Blue viability dye (Thermo Fisher) and underwent surface staining for CD4-BV510, CD25-BV605, PD-1-AF647, CD39-APC, CD73-PE, CTLA-4-PE-Cy7, GITR-PE-Cy5, and CD40L-APC-Cy7 (**Table 1**). The cells were subsequently permeabilized as described above and stained with FOXP3-AF488 and Helios-Pacific Blue before flow cytometric assessment on a Cytek Aurora 5L (16UV-16V-14B-10YG-8R) spectral flow cytometer and analyzed in FlowJo version 10.6.1 Software.

### Treg Suppression Assays

Post-expansion CD4^+^CD25^+^CD226^-^ Tregs and CD4^+^CD25^+^CD127^lo/-^ Tregs were collected on day 14 and immediately labeled with cell proliferation dye (CPD-eFluor 670; Biolegend), while autologous PBMCs were labeled with CTV as recommended by the manufacturers’ protocol. Tregs were co-cultured with PBMCs (Treg : PBMC ratios of 1:1, 1:2, 1:4, 1:8, 1:16, 1:32) in the presence of soluble anti-CD3 (8 µg/mL, Clone OKT3) and soluble anti-CD28 (4 µg/mL, Clone CD28.2) in triplicate for 96 hours. Replicates were pooled, subjected to surface staining for CD4-BV510 and CD8-PE-CF594 (**Table 1**), assessed using a Cytek Aurora 5L (16UV-16V-14B-10YG-8R) spectral flow cytometer, and analyzed in FlowJo version 10.6.1 Software. Percent suppression of responder cells was established by the division index (DI) method using proliferation modeling (40).

### Statistical Analysis

Generation of figures and statistical analysis were conducted using GraphPad Prism version 9.2.0 (GraphPad Software, San Diego, CA, USA). Data were analyzed by two-way ANOVA with Bonferroni’s *post hoc* test for multiple testing correction unless otherwise stated. Area under the curve (AUC) values were compared using paired t-tests (41). The p-value ≤ 0.05 was considered significant.

## Results

### CD25 Expression is Elevated on CD4^+^CD25^+^CD226^-^ Tregs

**Figure 2:**
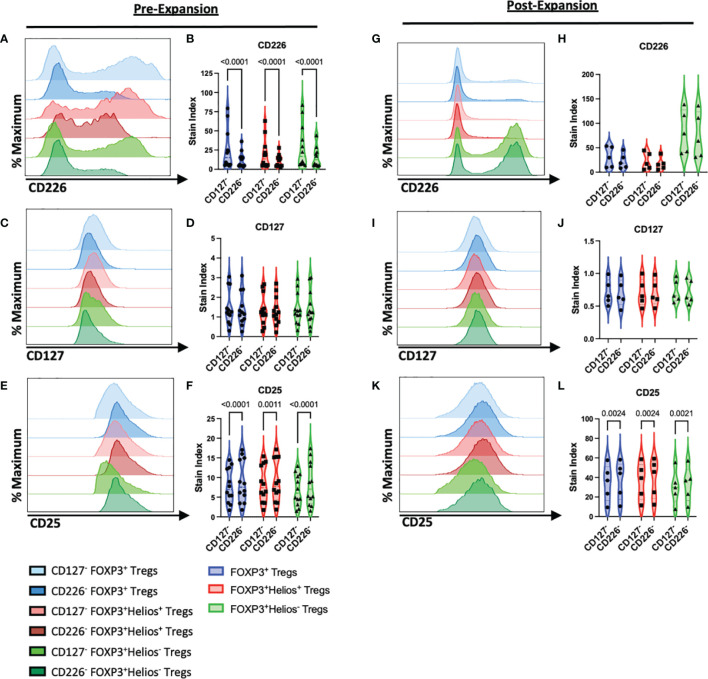
CD4^+^CD25^+^CD226^-^ Tregs Exhibit Increased CD25 Expression. Representative histograms show expression of cell surface markers on CD127^lo/-^ (lighter blue) and CD226^-^ CD4^+^CD25^+^FOXP3^+^ Tregs (darker blue), CD127^lo/-^ (lighter red) and CD226^-^ CD4^+^CD25^+^FOXP3^+^Helios^+^ Tregs (darker red), and CD127^lo/-^ (lighter green) and CD226^-^ CD4^+^CD25^+^FOXP3^+^Helios^-^ Tregs (darker green) with violin plots showing stain index (SI) fold change from FMO controls, **(A–F)** prior to expansion (*n* =12 biological with *n* = 2 technical replicates) and **(G–L)** following 14 days of *ex vivo* expansion (*n* = 5 biological with *n* = 2 technical replicates). **(A, B, G, H)** CD226, **(C, D, I, J)** CD127, **(E, F, K, L)** CD25. Significant P-values are reported on the figure for two-way ANOVA with Bonferroni correction for multiple comparisons of Treg isolation conditions from matched subjects.

To characterize the efficacy of the sorting strategies in isolating CD4^+^CD25^+^CD226^-^ versus CD4^+^CD25^+^CD127^lo/-^ Tregs (**Figure 1**), we examined surface expression levels of CD226, CD127, and CD25 on CD4^+^CD25^+^FOXP3^+^ total Tregs, FOXP3^+^Helios^+^ Tregs, as well as FOXP3^+^Helios^-^ Tregs by flow cytometry (**Figure S1**), both prior to and following *ex vivo* expansion (42). As expected, CD226 expression was significantly lower on CD4^+^CD25^+^CD226^-^ sorted cells, including total FOXP3^+^ Tregs (0.49-fold, p<0.0001) as well as FOXP3^+^Helios^+^ (0.55-fold, p<0.0001) and FOXP3^+^Helios^-^ subsets (0.60-fold, p<0.0001; **Figures 2A, B**). Prior to expansion, CD4^+^CD25^+^CD226^-^ and CD4^+^CD25^+^CD127^lo/-^ sorted Tregs displayed comparably low CD127 expression (**Figures 2C, D**). Yet, the CD4^+^CD25^+^CD226^-^ isolation strategy yielded a significantly higher CD25 gMFI on Tregs (1.13-fold, p<0.0001), including both the FOXP3^+^Helios^+^ (1.08-fold, p=0.0011) and FOXP3^+^Helios^-^ subsets (1.22-fold, p<0.0001; **Figures 2E, F**). As a result of expansion, CD4^+^CD25^+^CD226^-^ sorted Tregs re-expressed CD226 at similar levels to CD4^+^CD25^+^CD127^lo/-^ Tregs following *ex vivo* expansion, with the most dramatic upregulation of CD226 occurring in the FOXP3^+^Helios^-^ fraction (**Figures 2G, H**). However, CD127 levels remained comparably low across all Treg subsets (**Figures 2I, J**), and CD25 levels remained augmented on CD4^+^CD25^+^CD226^-^ versus CD4^+^CD25^+^CD127^lo/-^ sorted cells, including total Tregs (1.08-fold, p=0.0024), FOXP3^+^Helios^+^ Tregs (1.08-fold, p=0.0024) and FOXP3^+^Helios^-^ Tregs (1.10-fold, p=0.0021; **Figures 2K, L**).

### CD4^+^CD25^+^CD226^-^ Tregs Maintain Higher Purity and Lineage Stability

**Figure 3:**
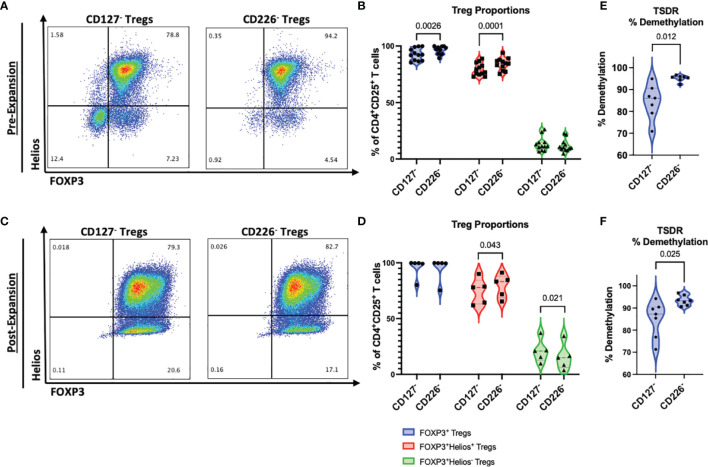
CD226^-^ Tregs maintain higher purity than conventionally sorted CD127^lo/-^ Tregs. Tregs from each FACS method were examined for FOXP3 and Helios expression **(A, B)** at day 0 following isolation (*n* = 12 biological with *n* = 2 technical replicates) and **(C, D)** at day 14 following *ex vivo* expansion (*n* = 5 biological with *n* = 2 technical replicates). **(A, C)** Representative flow plots pre-gated on live CD4^+^CD25^+^ cells show percentages of Treg subsets for CD127^lo/-^ sorted Tregs and CD226^-^ sorted Tregs. **(B, D)** Percentages of CD4^+^CD25^+^FOXP3^+^ Tregs (blue), CD4^+^CD25^+^FOXP3^+^Helios^+^ Tregs (red), and CD4^+^CD25^+^FOXP3^+^Helios^-^ Tregs (green) per FACS isolation method. P-values are reported on the figure for two-way ANOVA with Bonferroni *post-hoc* correction for multiple comparisons of Treg isolation conditions from matched subjects. **(E, F)** Percent demethylation at the TSDR of CD127^lo/-^ and CD226^-^ sorted Treg cultures, **(E)** pre-expansion and **(F)** post-expansion. Significant P-values are reported on the figure for paired T-tests, *n* = 7 biological with *n* = 2 technical replicates.

We previously identified CD4^+^CD25^+^CD127^lo/-^CD226^+^ Tregs as a subset with a higher proportion of IFNγ-producing pTregs, as compared to CD4^+^CD25^+^CD127^lo/-^CD226^-^ Tregs (30). To evaluate the potential of using a CD4^+^CD25^+^CD226^-^ sort for isolation of more lineage stable Tregs, as compared to the typical CD4^+^CD25^+^CD127^lo/-^ strategy, we examined the expression of the canonical Treg lineage-defining transcription factors, FOXP3 and Helios, using flow cytometry (**Figure S1**). We identified significantly increased percentages of FOXP3^+^ Tregs prior to expansion (+3.60%, p=0.0026), including an increased proportion of FOXP3^+^Helios^+^ Tregs (+4.70%, p=0.0001) within the CD4^+^CD25^+^CD226^-^ sorted population, compared to CD4^+^CD25^+^CD127^lo/-^ (**Figures 3A, B**). Importantly, these differences were not related to variations in donor sex (**Figure S2A**) or age (**Figure S2B**); though, we did identify a significantly non-zero slope (p=0.023) suggesting that the isolation of CD127^lo/-^ FOXP3^+^Helios^+^ Tregs increased with age for our data set (**Figure S2B**). Following a 14-day expansion period, significant increases in the FOXP3^+^Helios^+^ Treg (+3.57%, p=0.043) and decreases in the FOXP3^+^Helios^-^ Treg (-4.43%, p=0.021) subpopulations were observed from CD4^+^CD25^+^CD226^-^ versus CD4^+^CD25^+^CD127^lo/-^ sorted cells, despite comparable frequencies of total Tregs (**Figures 3C, D**). These data suggest that isolation of CD4^+^CD25^+^CD226^-^ Tregs may yield a more stable Treg population after *ex vivo* expansion, without compromising post-expansion yield (**Figure S3**).

tTregs display a distinct epigenetic profile, including the selective demethylation of the FOXP3-TSDR region (39). We therefore evaluated levels of TSDR methylation by RT-PCR. This analysis showed increased levels of TSDR demethylation in the CD4^+^CD25^+^CD226^-^ Treg population both before (+10.69%, p=0.012) and following expansion (+8.46%, p=0.025) compared to Tregs isolated by the CD4^+^CD25^+^CD127^lo/-^ marker profile (**Figures 3E, F**). These data corroborate our flow cytometry results identifying a higher purity of lineage stable Tregs in CD4^+^CD25^+^CD226^-^sorted cells (**Figures 3A–D**). Together, these results demonstrate high purity and lineage stability of CD4^+^CD25^+^CD226^-^ Tregs throughout *ex vivo* expansion.

### CD4^+^CD25^+^CD226^-^ Tregs Display a More Naïve Phenotype

**Figure 4:**
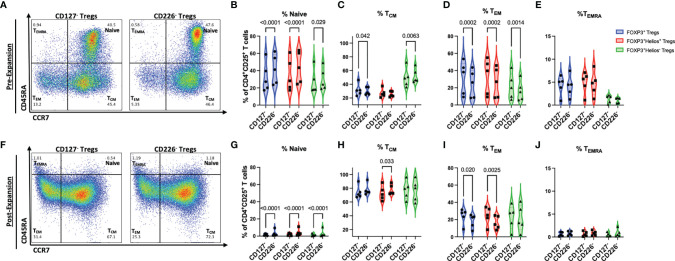
CD226^-^ Tregs display a more immunoregulatory phenotype than CD127^lo/-^ Tregs. Memory differentiation of Tregs from each FACS method was assessed by CD45RA and CD197 (CCR7) expression **(A–E)** at day 0 following isolation and **(F–J)** at day 14 following *ex vivo* expansion (*n* = 5 biological with *n* = 2 technical replicates). **(A, F)** Representative flow plots pre-gated on live CD4^+^CD25^+^ cells show percentages for each T cell memory subset for CD127^lo/-^ sorted Tregs and CD226^-^ sorted Tregs. Relative proportions of **(B, G)** CD45RA^+^CCR7^+^ naïve, **(C, H)** CD45RA^-^CCR7^+^ T_CM_, **(D, I)** CD45RA^-^CCR7^-^ T_EM_, and **(E, J)** CD45RA^+^CCR7^-^ T_EMRA_ subsets from CD127^lo/-^ sorted Treg and CD226^-^ sorted Treg cultures after gating on CD4^+^CD25^+^ FOXP3^+^ Tregs (blue), CD4^+^CD25^+^FOXP3^+^Helios^+^ Tregs (red), and CD4^+^CD25^+^FOXP3^+^Helios^-^ Tregs (green). P-values are reported on the figure for two-way ANOVA with Bonferroni *post-hoc* correction for multiple comparisons of Treg isolation conditions from matched subjects.

We next sought to assess the extent of differentiation in CD4^+^CD25^+^CD226^-^ and CD4^+^CD25^+^CD127^lo/-^ Tregs, pre- and post-expansion. Before expansion, CD4^+^CD25^+^CD226^-^ Tregs were found to contain significantly more naïve total Tregs (+8.92%, p<0.0001), FOXP3^+^Helios^+^ Tregs (+8.58%, p<0.0001), and FOXP3^+^Helios^-^ Tregs (+2.95%, p=0.029), as well as fewer T_CM_ total Tregs (-1.78%, p=0.042), yet T_CM_ FOXP3^+^Helios^-^ Treg frequencies were increased versus CD4^+^CD25^+^CD127^lo/-^ Tregs (+2.54%, p=0.0063, **Figures 4A–C**). Pre-expansion CD4^+^CD25^+^CD226^-^ Tregs also comprised fewer T_EM_ total Tregs (-6.97%, p=0.0002), FOXP3^+^Helios^+^ Tregs (-6.75%, p=0.0002), FOXP3^+^Helios^-^ Tregs (-5.21%, p=0.0014), with no significant differences in T_EMRA_ compared to CD4^+^CD25^+^CD127^lo/-^ Tregs (**Figures 4D, E**). Additionally, CD4^+^CD25^+^CD226^-^ cells represented a significantly greater proportion (1.89-fold, p=0.019) and absolute cell count (p=0.037) within the CD4^+^CD25^+^CD127^lo/-^ sorted Treg population, as compared to CD4^+^CD25^+^CD127^lo/-^CD45RA^+^ cells (**Figure S4**), suggesting that CD226^-^ enrichment for naïve and T_CM_ cells does not compromise post-sort yield as drastically as a four-marker CD4^+^CD25^+^CD127^lo/-^CD45RA^+^ naïve Treg isolation strategy (43, 44).

Post-expansion, higher percentages of remaining naïve Tregs in the total Treg (+1.47%, p<0.0001), FOXP3^+^Helios^+^ (+1.79%, p<0.0001), and FOXP3^+^Helios^-^ (+1.58%, p<0.0001) subpopulations, as well as higher percentages of T_CM_-differentiated FOXP3^+^Helios^+^ Tregs (+4.96%, p=0.033) were observed in the CD4^+^CD25^+^CD226^-^ sorted Tregs as compared to CD4^+^CD25^+^CD127^lo/-^ sorted cells (**Figures 4F–H**). Additionally, CD4^+^CD25^+^CD226^-^ Treg expansion yielded significantly lower percentages of T_EM_-differentiated total Tregs (-5.63%, p=0.020) and FOXP3^+^Helios^+^ Tregs (-8.05%, p=0.0025), with no differences in T_EMRA_-differentiated Tregs observed post-expansion (**Figures 4I, J**). Collectively, these data support the use of CD4^+^CD25^+^CD226^-^ for the isolation of naïve Tregs which differentiate more readily into a T_CM_ as opposed to T_EM_ phenotype after expansion. This finding has potential implications for Treg ACT in settings of autoimmunity and transplantation: specifically, the preferential outgrowth of T_CM_ from CD226^-^ Tregs may lead to better engraftment efficiency and localization to secondary lymphoid organs where autoimmune priming and graft versus host disease (GvHD) are initiated (45).

### CD4^+^CD25^+^CD226^-^ Tregs Display a More Immunoregulatory Cytokine Profile

**Figure 5:**
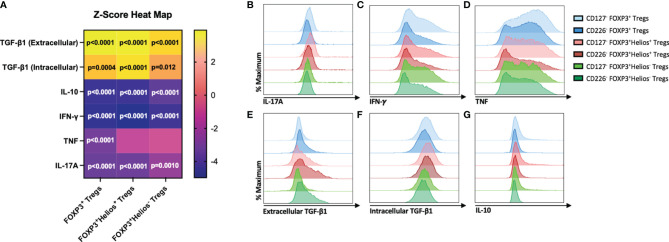
CD226^-^ Tregs exhibit a less inflammatory cytokine profile than CD127^lo/-^ Tregs. Cytokine production by 14-day *ex vivo* expanded CD127^lo/-^ sorted Treg and CD226^-^ sorted Treg cultures was examined by flow cytometry following a four-hour PMA/Ionomycin stimulation in the presence of a protein transport inhibitor. **(A)** Heat map shows the mean fold change of protein expression (z-score) of cytokines (rows) by CD226^-^ FOXP3^+^, FOXP3^+^Helios^+^, and FOXP3^+^Helios^-^ Treg populations (columns) compared to CD127^lo/-^ Treg populations. *n* = 9 biological with *n* = 2 technical replicates. Significant P-values are reported on the figure for two-way ANOVA with Bonferroni’s multiple comparisons between Treg isolation conditions of matched subjects. Representative histograms show expression of **(B)** IL-17A, **(C)** IFN-γ, **(D)** TNF, **(E)** Extracellular TGF-β1, **(F)** Intracellular TGF-β1, and **(G)** IL-10 for CD127^lo/-^ (lighter blue) and CD226^-^ CD4^+^CD25^+^FOXP3^+^ Tregs (darker blue), CD127^lo/-^ (lighter red) and CD226^-^ CD4^+^CD25^+^FOXP3^+^Helios^+^ Tregs (darker red), and CD127^lo/-^ (lighter green) and CD226^-^ CD4^+^CD25^+^FOXP3^+^Helios^-^ Tregs (darker green).

The production of pro-inflammatory cytokines is a hallmark of Treg instability, which may contribute to a loss of immune tolerance in autoimmune disorders (30, 46). Therefore, we sought to determine whether CD4^+^CD25^+^CD226^-^ Tregs possess a more immunoregulatory cytokine profile than CD4^+^CD25^+^CD127^lo/-^ Tregs. Flow cytometric assessment of cytokine production in unstimulated cells revealed no differences in cytokine production at rest (**Figure S5**), but following PMA/Ionomycin stimulation, we observed decreased pro-inflammatory IL-17A expression by CD4^+^CD25^+^CD226^-^ sorted Tregs within the total Treg (0.92-fold, p<0.0001), FOXP3^+^Helios^+^ (0.92-fold, p<0.0001), and FOXP3^+^Helios^-^ (0.94-fold, p=0.0010) populations (**Figures 5A, B**). Additionally, significant decreases were observed in IFN-γ expression by CD4^+^CD25^+^CD226^-^ sorted total Tregs (0.86-fold, p<0.0001), FOXP3^+^Helios^+^ (0.85-fold, p<0.0001), and FOXP3^+^Helios^-^ (0.87-fold, p<0.0001) as well as TNF expression by CD4^+^CD25^+^CD226^-^ sorted total Tregs (0.80-fold, p<0.0001), both of which are pro-inflammatory cytokines associated with a Th1 effector profile (**Figures 5A, C, D**) (47, 48). We found significantly increased expression of both extracellular and intracellular TGF-β1 by CD4^+^CD25^+^CD226^-^ sorted total Tregs (1.87-fold, p<0.0001; 1.15-fold, p=0.0004, respectively), FOXP3^+^Helios^+^ Tregs (1.55-fold, p<0.0001; 1.16-fold, p<0.0001), and FOXP3^+^Helios^-^ Tregs (1.56-fold, p<0.0001; 1.10-fold, p=0.012) (**Figures 5A, E, F**). Given that TGF-β1 is critical for inhibiting Th1 differentiation (49, 50), these findings corroborate the observations of decreased pro-inflammatory cytokine expression by CD4^+^CD25^+^CD226^-^ Tregs. Interestingly, expression of the anti-inflammatory cytokine IL-10 was significantly decreased in CD4^+^CD25^+^CD226^-^ sorted total Tregs (0.83-fold, p<0.0001), FOXP3^+^Helios^+^ Tregs (0.83-fold, p<0.0001), and FOXP3^+^Helios^-^ Tregs (0.85-fold, p<0.0001) (**Figures 5A, G**). IL-10, however, is commonly produced by Tr1-like T cells, which differentiate from conventional CD4^+^ T cells and may only transiently express FOXP3 (2), providing a potential mechanism for its decreased production. This observation is consistent with CD4^+^CD25^+^CD226^-^ T cell sorting resulting in a decreased frequency of effector subsets. Overall, these data suggest that CD4^+^CD25^+^CD226^-^ Tregs maintain a more immunoregulatory cytokine profile than the CD4^+^CD25^+^CD127^lo/-^ counterpart.

### CD4^+^CD25^+^CD226^-^ Tregs Present Increased Surface Expression of PD-1 and CD39 Following Activation

**Figure 6:**
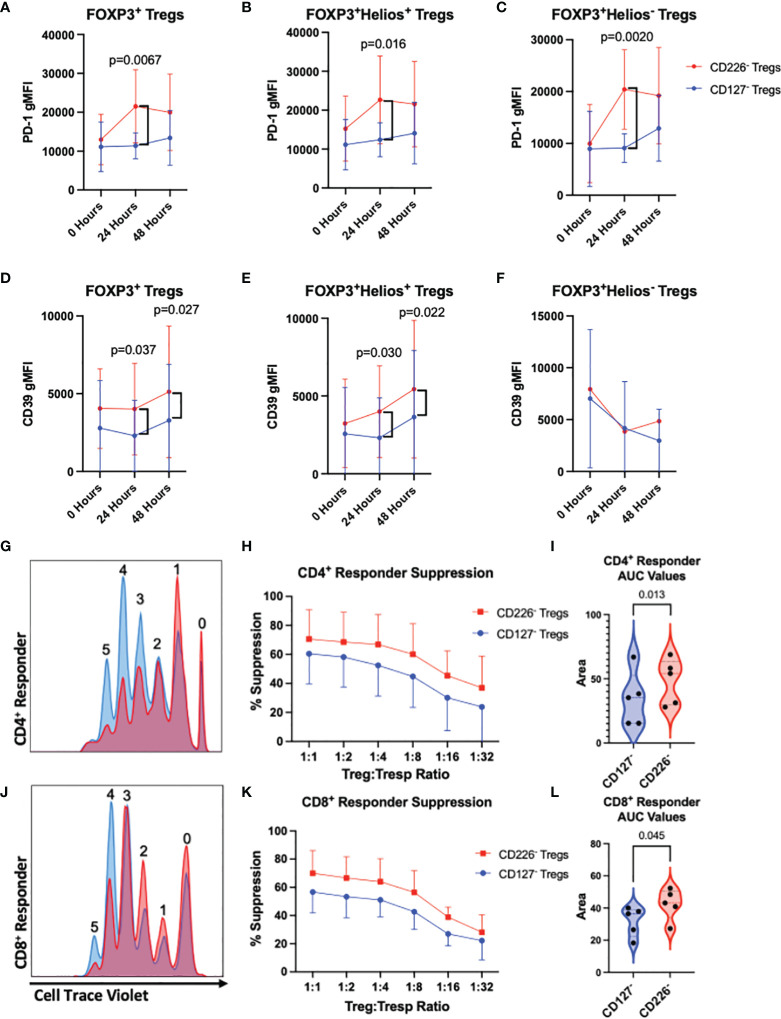
CD226^-^ Tregs demonstrate an increased suppressive phenotype and *ex vivo* suppressive capabilities as compared to CD127^lo/-^ Tregs. **(A–C)** PD-1 and **(D–F)** CD39 expression was assessed by flow cytometry on total FOXP3^+^ Tregs **(A, D)**, FOXP3^+^Helios^+^ Tregs **(B, E)**, and FOXP3^+^Helios^-^ Tregs **(C, F)** from 14-day *ex vivo* expanded CD127^lo/-^ sorted Tregs versus CD226^-^ Tregs following co-culture with autologous PBMCs in the presence of soluble α-CD3 and α-CD28 for 0, 24, or 48 hours. *n* = 5 biological replicates. Significant P-values reported on the figure for two-way ANOVA with Bonferroni’s multiple comparison between Treg isolation conditions of matched subjects. CD127^lo/-^ sorted Treg (blue) and CD226^-^ sorted Treg cultures (red) were by expanded for 14-days *ex vivo*, then labeled with Cell Proliferation Dye eFluor670 and co-cultured in decreasing two-fold dilutions with CellTrace Violet-labeled autologous responder PBMCs in the presence of soluble α-CD3 and α-CD28 antibodies for four days before flow cytometric assessment of **(G–I)** CD4^+^ and **(J–L)** CD8^+^ T cell proliferation. **(G, J)** Representative dye dilution plots demonstrating suppression of responders. Percent suppression of **(H)** CD4^+^ and **(K)** CD8^+^ responders was quantified by the division index method, and comparisons between FACS conditions were made using **(I, L)** area under the curve (AUC) values for each percent suppression curve (40, 41). Data reflects *n* = 5 biological with *n* = 3 technical replicates. Significant P-values are reported on the figure for paired T-tests comparing Treg isolation conditions from matched subjects.

To identify whether CD4^+^CD25^+^CD226^-^ sorted Tregs demonstrate differential expression of proteins associated with Treg-mediated suppression, we evaluated PD-1, CD39, CD73, CD40L, GITR, and CTLA-4 by flow cytometry on Tregs expanded from CD226^-^ and CD127^lo/-^ preparations, prior to and following activation by autologous PBMCs. After 24 hours of stimulation, CD226^-^ sorted Tregs displayed increased expression of PD-1 on both total FOXP3^+^ Tregs (1.89-fold, p=0.0067) as well as within the FOXP3^+^Helios^+^ Treg (1.82-fold, p=0.016) and FOXP3^+^Helios^-^ Treg subsets (2.24-fold, p=0.0020) as compared to CD127^lo/-^ sorted counterparts; however, there were no significant differences observed at baseline or after 48 hours of stimulation (**Figures 6A–C**). Furthermore, CD226^-^ Tregs exhibited significantly increased expression of CD39 at 24 and 48 hours in both total Tregs (1.75-fold, p=0.037; 1.56-fold, p=0.027, respectively) and the FOXP3^+^Helios^+^ subset (1.73-fold, p=0.030; 1.47-fold, p=0.022, respectively) compared to CD127^lo/-^ Tregs (**Figures 6D–F**). Compared to CD127^lo/-^ sorted Tregs, CD226^-^ sorted Tregs did not exhibit any significant differences in surface expression of CD73, CD40L, GITR, or CTLA-4, including after stimulation (**Figure S6**). Taken together, these data suggest that CD226^-^ Tregs may exhibit enhanced suppression of responder T cells *via* the PD-1/PD-L1 and CD39/CD73 ectonucleotidase pathways (51, 52).

### CD4^+^CD25^+^CD226^-^ Tregs Demonstrate Increased *Ex Vivo* Suppressive Capabilities

During expansion, Treg cultures can be prone to lineage instability as well as outgrowth of Tconv contaminants, ultimately impacting therapeutic potential by reducing suppressive capabilities (53). Given that CD4^+^CD25^+^CD226^-^ sorted Tregs exhibited a greater frequency of lineage stable FOXP3^+^Helios^+^ Tregs than CD4^+^CD25^+^CD127^lo/-^ Tregs, corroborated by epigenetic, differentiation and cytokine profile data, we sought to understand how sorting method might impact suppressive capacity. To accomplish this, we conducted dual-color *ex vivo* suppression assays using serial dilutions of expanded Tregs (sorted *via* CD4^+^CD25^+^CD127^lo/-^ or CD4^+^CD25^+^CD226^-^) with autologous whole PBMC responder cells. CD226^-^ sorted Tregs exhibited significantly increased suppression of both CD4^+^ and CD8^+^ effector T cell subsets, as demonstrated by decreased division indices for CTV labeled responder cells, as compared to those observed in CD127^lo/-^ sorted Treg cocultures (**Figures 6G–L**). Importantly, we did not observe dilution of CPD in either Treg population, suggesting that differences in suppression were not due to further expansion of CD226^-^ sorted Tregs. These data suggest that isolation of CD4^+^CD25^+^CD226^-^ Tregs yields a more suppressive Treg population that may improve ACT efficacy compared to conventional CD4^+^CD25^+^CD127^lo/-^ Tregs.

## Discussion

The requirement for long-term stability following engraftment in Treg-ACT necessitates the use of a combination of surface markers that function as a surrogate for the tTreg lineage-defining transcription factors. In this study, we compared the commonly employed Treg FACS isolation method using the CD4^+^CD25^+^CD127^lo/-^ marker profile to an alternative CD4^+^CD25^+^CD226^-^ approach. Importantly, sorted cells were assessed both pre- and post-expansion with the latter rested for 7 days prior to phenotypic, epigenetic, and functional characterization in order to mitigate transient upregulation of Helios in pTreg and contaminating Tconv (54) as a potential confounding factor. The resulting data demonstrate that isolation of CD4^+^CD25^+^CD226^-^ Tregs yields a greater frequency of lineage stable FOXP3^+^Helios^+^ Tregs, a reduced percentage of FOXP3^+^Helios^-^ Tregs, and increased demethylation at the *FOXP3* TSDR compared to CD4^+^CD25^+^CD127^lo/-^ Tregs. Among total Treg, FOXP3^+^Helios^+^ Treg and FOXP3^+^Helios^-^ Treg subsets, increased expression of CD25 was observed on CD4^+^CD25^+^CD226^-^ sorted cells without compromising the low CD127 expression typically achieved using the CD4^+^CD25^+^CD127^lo/-^ strategy. Together, this suggests that CD4^+^CD25^+^CD226^-^ Tregs may have a higher avidity for IL-2, which is putatively reported to result in downstream pSTAT5-signaling to reinforce lineage stability, fitness, and function (55–57). While the increase in purity we observed is modest relative to CD4^+^CD25^+^CD127^lo/-^ Tregs, we expect that these small improvements in initial purity may have a significant biological impact on the long-term survival and stability of a transferred population in ACT applications (58).

It is important to note that the data herein were derived from PBMC samples from healthy subjects (i.e., the general population). We speculate that the differences observed between CD4^+^CD25^+^CD127^-^ and CD4^+^CD25^+^CD226^-^ Tregs may be more prominent in autoimmune subjects, particularly during periods of acute inflammation where IL-2R signaling defects have been observed (59–61). This concept is critical when considering the transfer of islet antigen-specific Tregs created using genetically-modified T cell receptors (TCR) or chimeric antigen receptors (CAR) that could potentially become directly pathogenic toward islets and/or β-cells in situations of Treg instability (62–64).

To further characterize CD4^+^CD25^+^CD226^-^ Tregs, we assessed T cell memory differentiation markers and found increased proportions of naïve Tregs both before and after expansion, along with reduced proportions of effector memory Tregs post-expansion as compared to the traditional CD4^+^CD25^+^CD127^lo/-^ strategy. These results suggest that CD4^+^CD25^+^CD226^-^ may not only serve as a better set of markers to identify lineage stable Tregs but also, to avoid effector contaminants. This notion is supported by prior work by Hoffmann and colleagues who initially demonstrated that CD45RA^+^ naïve Tregs displayed increased stability upon expansion as compared to CD45RA^-^ memory Tregs (65), a finding that we have consistently replicated from both umbilical cord and adult peripheral blood samples (13, 28, 66). Previous studies have identified the marker profile CD4^+^CD25^+^CD127^lo/-^CD45RA^+^ as selecting for predominately naïve tTregs; however, this isolation method yields a much smaller population than required for many ACT applications (43, 44). While our three-marker sorting strategy enriches for naïve Tregs, it also captures CD4^+^CD25^+^CD226^-^ memory Tregs, resulting in a greater FACS yield compared to a CD4^+^CD25^+^CD127^lo/-^CD45RA^+^ strategy. Hence, the CD4^+^CD25^+^CD226^-^ sorting strategy strikes a practical balance between the desire to enrich for naïve Tregs versus CD4^+^CD25^+^CD127^lo/-^ isolation but also, maximize cell yield as compared to CD4^+^CD25^+^CD127^lo/-^CD45RA^+^ isolation. Similarly, while TIGIT has been identified as a marker of lineage stable tTregs, we previously reported that TIGIT^+^ cells had a limited expansion capacity and therefore, would not produce enough Tregs for ACT (30, 31). Importantly, we observed a significantly lower proportion of T_EM_-differentiated Tregs and a higher proportion of T_CM_-differentiated Tregs expanded *ex vivo* from the CD226^-^ Treg population. This observation suggests that CD4^+^CD25^+^CD226^-^ Tregs may not only be longer-lived, but potentially, localize more readily in secondary lymphoid organs (17). This remains a critical issue for Treg-ACT applications in the context of T1D, as Tregs must be able to migrate to sites of inflammation and priming, specifically to the pancreatic draining lymph nodes where recent studies have revealed the presence of a stem-cell like CD8^+^ T cell progenitor population that significantly contributes to pancreatic β-cell destruction in the NOD model of T1D (67).

During our assessment of the therapeutic potential of this CD4^+^CD25^+^CD226^-^ Treg subset, we found decreased expression of the pro-inflammatory cytokines IL-17A, TNF, and IFN-γ, associated with Th17 and Th1 responses, in comparison to CD4^+^CD25^+^CD127^lo/-^ Tregs following PMA/Ionomycin stimulation. This finding is especially important in the context of autoimmunity, as Th1 and Th17 effectors have been associated with several autoimmune diseases, including T1D and MS, suggesting that CD4^+^CD25^+^CD226^-^ Treg isolation may potentially deplete these pathogenic Tregs and present reduced risk of pro-inflammatory ex-Treg outgrowth compared to CD4^+^CD25^+^CD127^lo/-^ Treg isolation (68–70). Beyond this, PMA/Ionomycin stimulated CD226^-^ sorted Tregs had increased intracellular and surface expression of the immunoregulatory molecule, TGF-β1 (71). Following TCR activation, CD226^-^ Tregs had increased expression of the immunoregulatory checkpoint molecules, PD-1 and CD39, which are associated with programmed cell death and ATP hydrolysis, respectively (52, 72). Inhibition or dysregulation of these checkpoint regulators has been associated with the development of autoreactivity (73, 74). Finally, given our observation that CD226^-^ sorted Tregs had higher CD25 expression post-expansion, it is possible that the augmented suppressive capacity observed could, at least in part, be related to increased competition for IL-2. Altogether, our data support the notion that differences in IL-2 consumption, cytokine production, and contact-dependent mechanisms may all contribute toward the increased level of suppression observed with CD226^-^ versus CD127^lo/-^ sorted Tregs.

Tregs are emerging as a powerful therapeutic modality in a broad array of autoimmune settings (75, 76). While our study supports CD226^-^ Tregs as a robust population to yield stable FOXP3^+^Helios^+^ Tregs, we note the limitation that our studies were conducted using general population control samples. Further research is needed to determine if CD226^-^ Tregs will provide increased purity and stability in patients with active autoimmune disease. Indeed, there are a number of outstanding questions regarding the TCR repertoire, homing receptors, and *in vivo* trafficking of CD226^-^ Tregs relative to CD127^lo/-^ Tregs (77).

These findings also raise a number of considerations regarding ACT with CD226^-^ Tregs and therapeutic targeting of the CD226 pathway in situations of autoimmunity. On one hand, our data related to CD226 being highly expressed on effector T cells supports additional studies targeting this pathway *in vivo* to block destructive autoimmunity. However, this approach should be taken with some caution, as we note that CD226 is also highly expressed by IL-10-secreting Tr1-like T cells (78). Thus, any immunotherapy seeking to inhibit CD226 on Tregs would likely need to be carefully dosed to increase Treg stability without compromising CD226-mediated Tr-1 like T cell function. Furthermore, these results support the continued investigation of the precise mechanisms by which reduced CD226 expression allows for increased Treg lineage stability and suppressive capacity. We note that additional studies are currently underway in our laboratory to assess the impact of CD226 on Tregs using both targeted biologics, along with global and conditional knockout approaches in animal models (37), as well as through gene targeting approaches in human Tregs. In summary, our findings present a novel method to generate a highly stable and suppressive Treg subset for use in ACT by initially eliminating Tregs expressing the costimulatory molecule, CD226.

## Data Availability Statement

The raw data supporting the conclusions of this article will be made available by the authors, without undue reservation.

## Author Contributions

MB: writing – original draft, writing – review and editing, formal analysis, visualization, validation, investigation, project administration, and supervision. LP: investigation, writing – review and editing, and funding acquisition. SH, JA, LS, KN, and EC: investigation and writing – review and editing. HS, CF, and AP: writing - review and editing. MS: writing - review and editing and funding acquisition. TB: conceptualization, writing – review and editing, funding acquisition, project administration, and supervision. TB is the guarantor of this work and, as such, had full access to all the data in the study and takes responsibility for the integrity of the data and the accuracy of the data analysis. All authors contributed to the article and approved the submitted version.

## Funding

Funding was provided by the National Institutes of Health through the support of grants to LDP (T32 DK108736) and to TMB (R01 DK106191, HIRN UG3/UH3 DK122638, P01 AI042288). Additional programmatic support was provided by Diabetes Research Connection to MS (DRC Project 45) and programmatic support by Leona M. and Harry B. The Helmsley Charitable Trust.

## Conflict of Interest

Author HS was employed in ROSALIND, Inc. Author CF was employed in NanoString Technologies, Inc. Authors TB, HS, and CF share intellectual property related to the use of CD226- Tregs for the treatment of autoimmune diseases.

The remaining authors declare that the research was conducted in the absence of any commercial or financial relationships that could be construed as a potential conflict of interest.

## Publisher’s Note

All claims expressed in this article are solely those of the authors and do not necessarily represent those of their affiliated organizations, or those of the publisher, the editors and the reviewers. Any product that may be evaluated in this article, or claim that may be made by its manufacturer, is not guaranteed or endorsed by the publisher.
