# Supplementary material for: Human CD4+CD25+CD226- Tregs Demonstrate Increased Purity, Lineage Stability, and Suppressive Capacity Versus CD4+CD25+CD127lo/- Tregs for Adoptive Cell Therapy
Source: Front Immunol. 2022 May 26;13:873560. doi: 10.3389/fimmu.2022.873560 (PMC9178079; doi:10.3389/fimmu.2022.873560)
Supplement: Supplementary file 1 [file DataSheet_1.docx]

# Supplemental Figures:

**
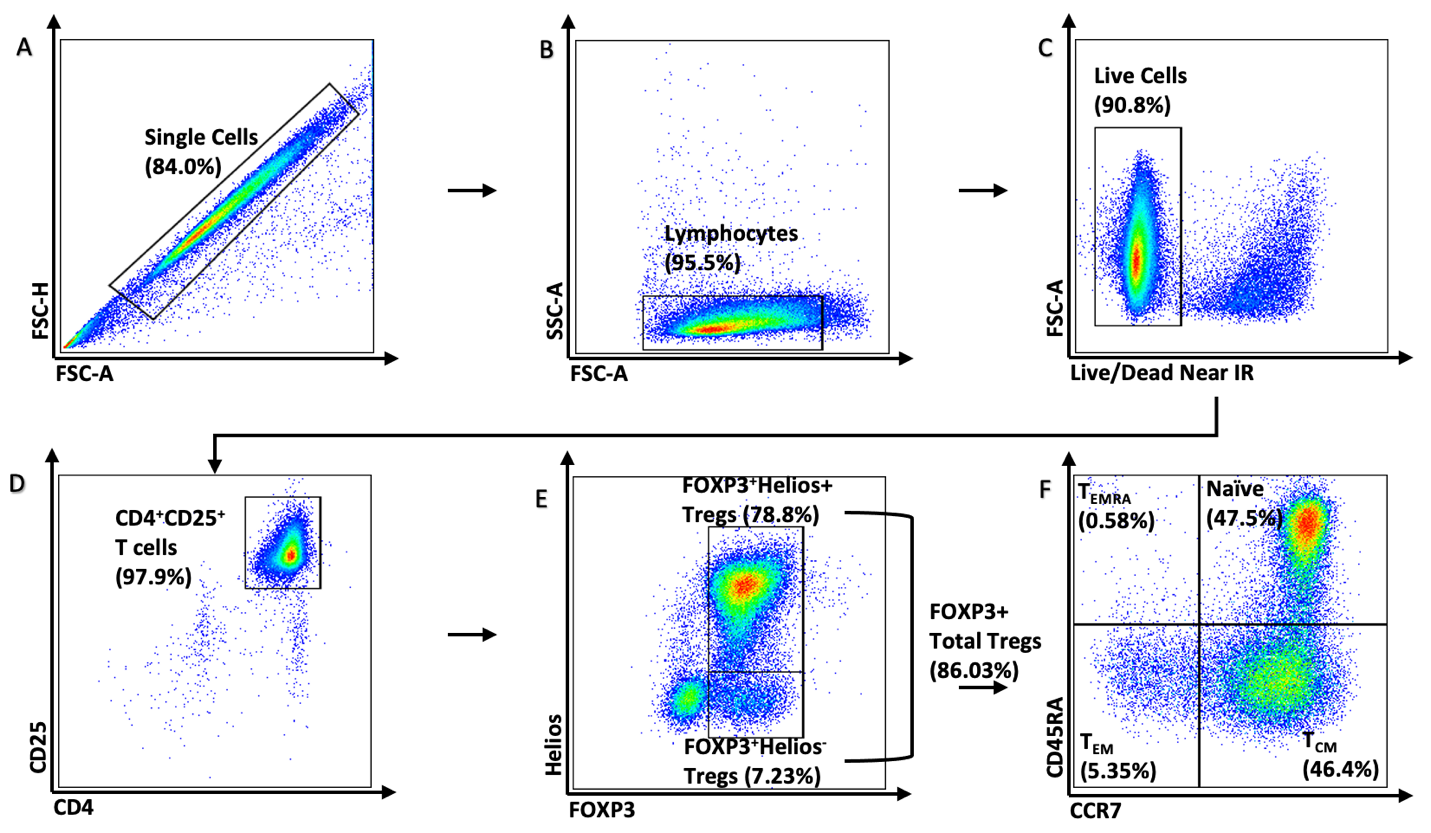
Supplemental Figure 1. Gating Strategy for Phenotyping of FOXP3^+^Helios^-^ and FOXP3^+^Helios^+^ Treg Subsets.** Representative flow plots demonstrate the method by which Total, FOXP3^+^Helios^+^, and FOXP3^+^Helios^-^ Treg subsets were defined from CD4^+^CD25^+^ T cells for subsequent assessment of memory differentiation and CD25/CD127/CD226 gMFI using a Cytek Aurora 3L Spectral Flow Cytometer. (**A**) Single cells were gated upon using a forward scatter area (FSC-A) by forward scatter height (FSC-H) plot. (**B**) Lymphocyte gating was accomplished using forward scatter area (FSC-A) by side scatter area (SSC-A). (**C**) Live lymphocytes were identified using a Live/Dead Near IR dye exclusion. (**D**) Within live CD4^+^CD25^+^ T cells, (**E**) total Tregs were defined as all live CD4^+^CD25^+^FOXP3^+^ cells, with additional Treg subpopulations gated as live CD4^+^CD25^+^FOXP3^+^Helios^+^ and CD4^+^CD25^+^FOXP3^+^Helios^-^ cells. (**F**) For total Tregs as well as FOXP3^+^Helios^-^ and FOXP3^+^Helios^+^ subsets, phenotypes were assessed as CD45RA^+^CCR7^+^ naïve, CD45RA^-^CCR7^+^ T_CM_, CD45RA^-^CCR7^-^ T_EM_, and CD45RA^+^CCR7^-^ T_EMRA_.

**
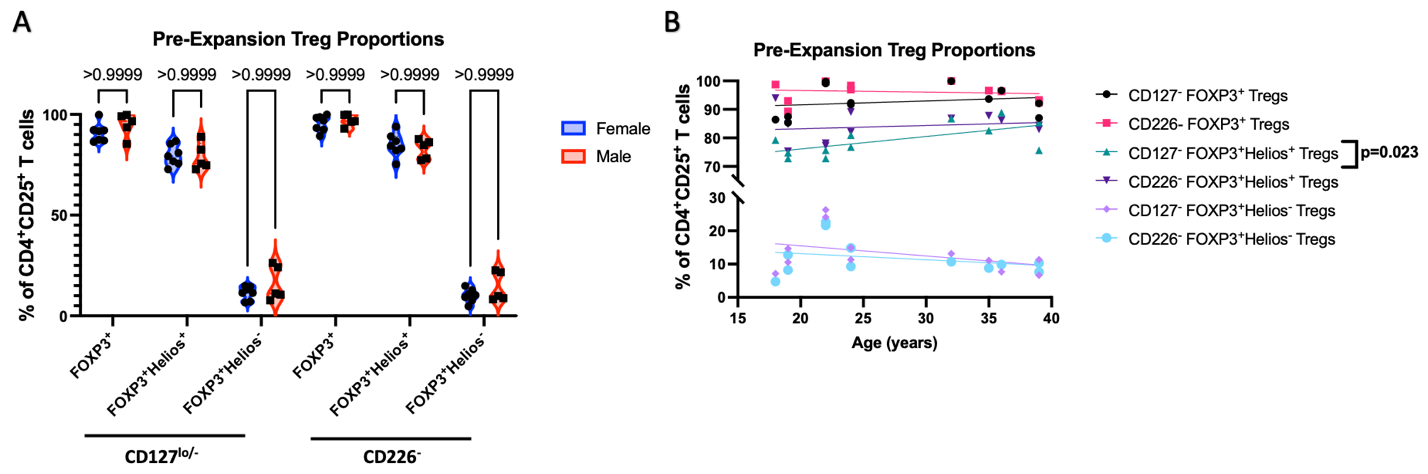
Supplemental Figure 2. Proportions of Treg Subsets following FACS Isolation and Association with Biological Sex or Age.** (**A**) Proportions of CD4^+^CD25^+^FOXP3^+^ Total, FOXP3^+^Helios^+^, and FOXP3^+^Helios^-^ Tregs were compared between females (blue, *n*=7) and males (red, *n*=5) for both the CD127^lo/-^ and CD226^-^ Treg isolation strategies. (**B**) Proportions of CD4^+^CD25^+^FOXP3^+^ Total Tregs, FOXP3^+^Helios^+^ Tregs, and FOXP3^+^Helios^-^ Tregs were compared as a function of age for both the CD127^lo/-^ and CD226^-^ Treg isolation strategies, with linear regression trendlines. A significant P-value representing statistical significance of the deviation of the slope from zero for CD127^-^ FOXP3^+^Helios^+^ Tregs is indicated on the figure.

**Supplemental Figure 3. Fold-Expansion of CD226^-^ Tregs Does Not Significantly Differ from CD4^+^CD25^+^CD127^lo/-^ Tregs.** Fold-Expansion of CD127^lo/-^ and CD226^-^ sorted Treg populations

are shown following 7 and 14 days of expansion. Data reflects *n*=7 biological replicates. Significant P-values are reported on the figure for one-way ANOVA with Bonferroni correction for multiple comparisons of Treg isolation conditions from matched subjects.**
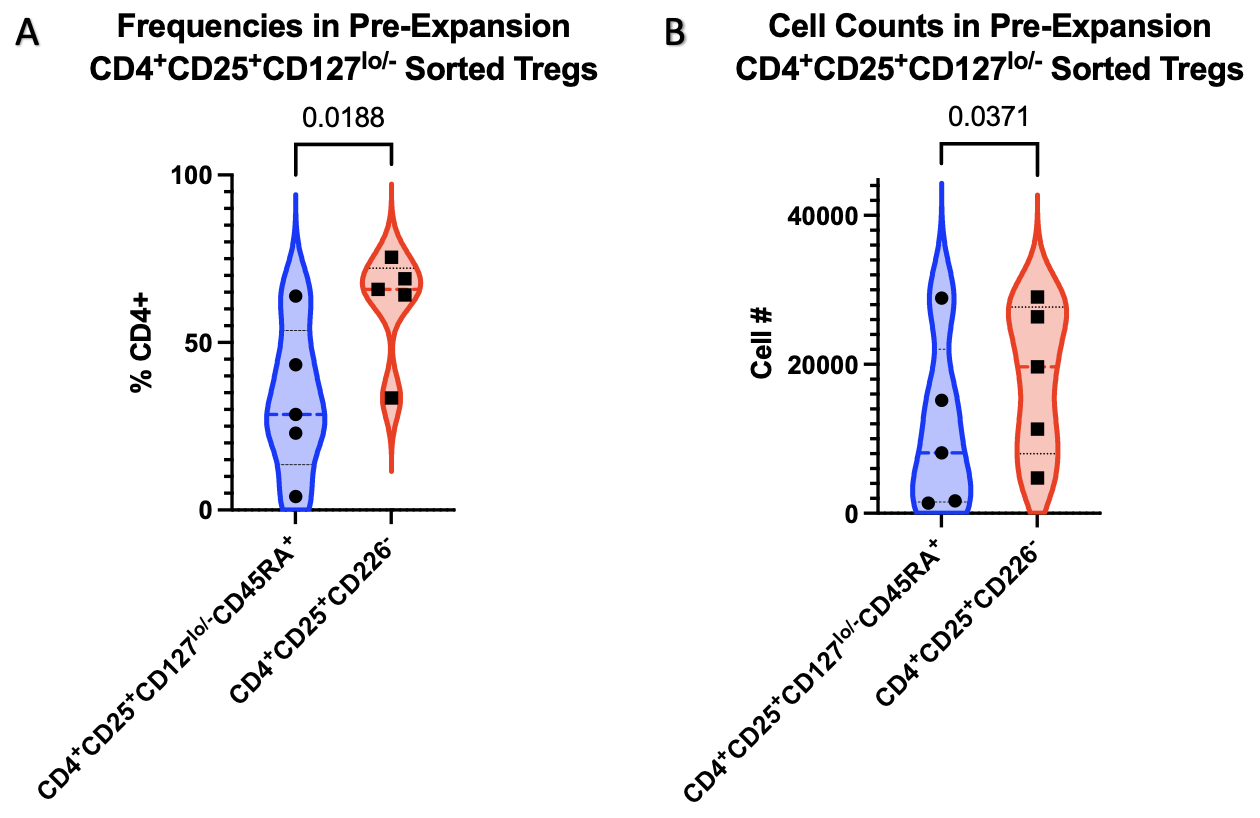
Supplemental Figure 4. CD226^-^ Tregs Represent a Greater Proportion of Sorted CD4^+^CD25^+^CD127^lo/-^ Tregs Compared to CD45RA^+^ Tregs.** (**A**) Proportions of CD4^+^ T cells and (**B**) cell counts of CD4^+^CD25^+^CD127^lo/-^CD45RA^+^ and CD4^+^CD25^+^CD226^-^ Tregs subpopulations within samples of 1 x 10^5^ pre-expansion CD4^+^CD25^+^CD127^lo/-^ sorted Tregs are shown. Data reflects *n*=5 biological replicates. Significant P-values are reported on the figure for paired T-tests comparing Treg subset frequencies and cell counts within samples.

**
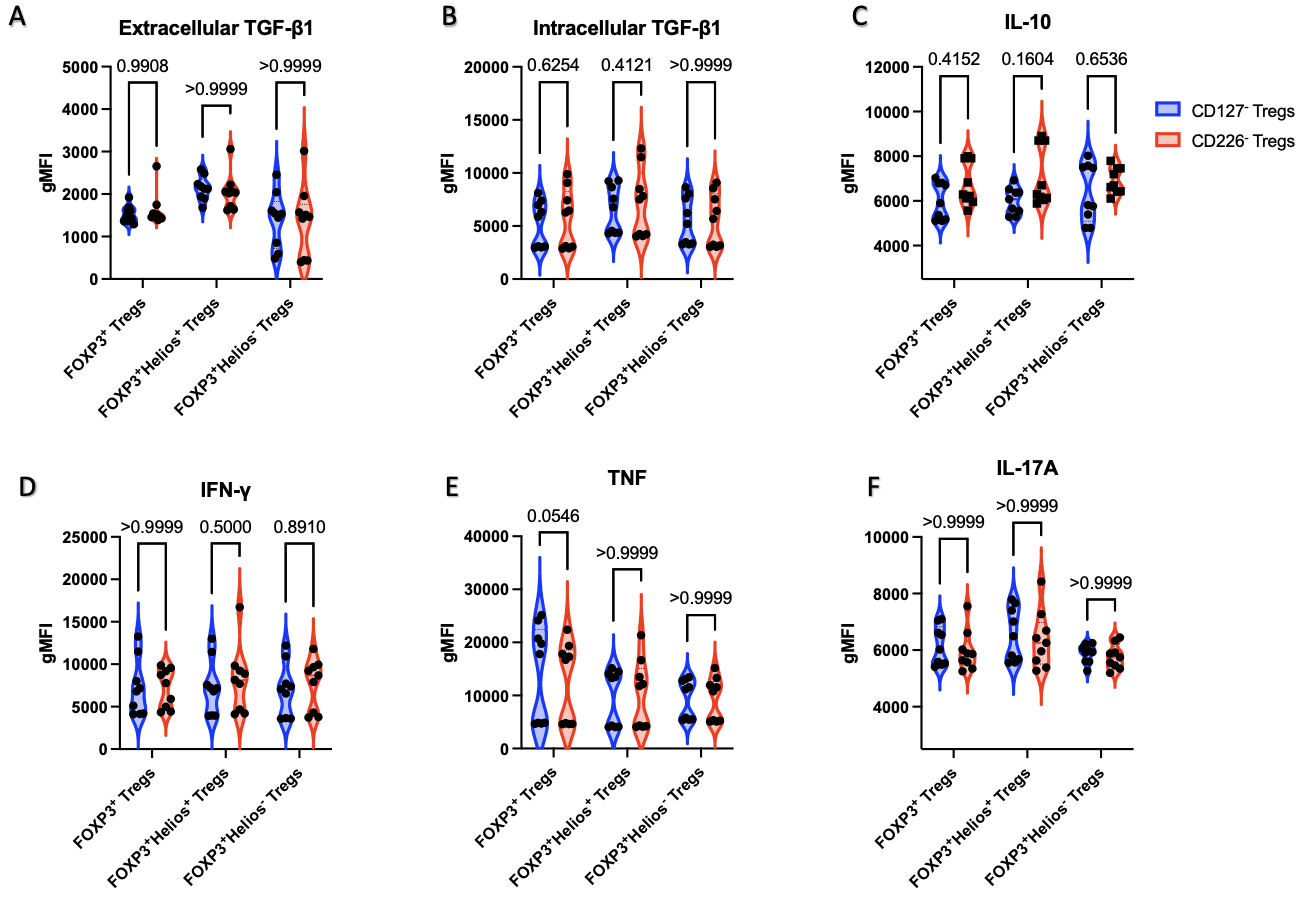
**Supplemental Figure 5. **Cytokine Profile of CD226^-^ Tregs Does Not Significantly Differ from CD127^lo/-^ Tregs at Rest.** Cytokine production by 14-day *ex vivo* expanded CD127^lo/-^ sorted Treg and CD226^-^ sorted Treg cultures was examined by flow cytometry without prior stimulation. (**A-F**) Violin plots show gMFIs of cytokines produced by unstimulated CD127^lo/-^ sorted Treg (blue) and CD226^-^ sorted Treg (red) cultures for FOXP3^+^ Tregs, FOXP3^+^Helios^+^ Tregs, and FOXP3^+^Helios^-^ Tregs. (**A**) Extracellular TGF-β1, (**B**) Intracellular TGF-β1, (**C**) IL-10, (**D**) IFN-γ, (**E**) TNF, (**F**) IL-17A. *n*=9 biological with *n*=2 technical replicates. All P-values were greater than 0.05 when using two-way ANOVA with Bonferroni post-hoc correction for multiple comparisons of Treg isolation conditions from matched subjects.

**
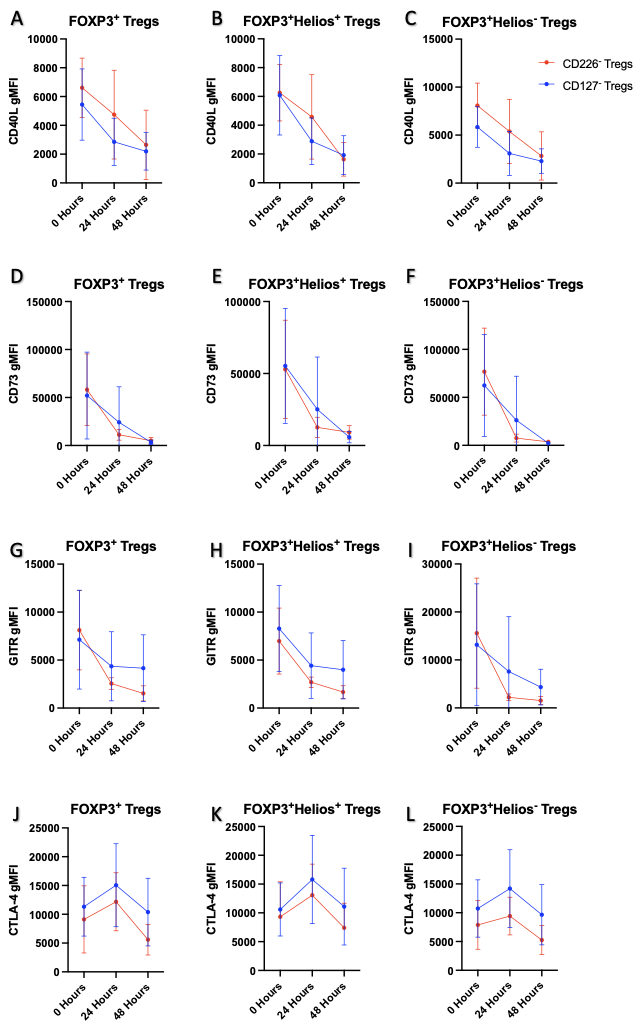
**

**Supplemental Figure 6. Expression of CD40L, CD73, GITR, and CTLA-4 on CD226^-^ Tregs Does Not Significantly Differ from CD4^+^CD25^+^CD127^lo/-^ Tregs Following Activation.** (**A-C**) CD40L, (**D-F**) CD73, (**G-I**) GITR, and (**J-L**) CTLA-4 expression was assessed by flow cytometry on total FOXP3^+^ Tregs (**A, D, G, J**), FOXP3^+^Helios^+^ Tregs (**B, E, H, K**), and FOXP3^+^Helios^-^ Tregs (**C, F, I, L**) from 14-day *ex vivo* expanded CD127^lo/-^ sorted Tregs versus CD226^-^ Tregs following co-culture with autologous PBMCs in the presence of soluble ⍺-CD3 and ⍺-CD28 for 0, 24, or 48 hours. *n*=5 biological replicates. All P-values were greater than 0.05 when using two-way ANOVA with Bonferroni post-hoc correction for multiple comparisons of Treg isolation conditions from matched subjects.
